# New insights into gelatinization mechanisms of cereal endosperm starches

**DOI:** 10.1038/s41598-018-21451-5

**Published:** 2018-02-14

**Authors:** Shujun Wang, Chen Chao, Fengjuan Xiang, Xiu Zhang, Shuo Wang, Les Copeland

**Affiliations:** 1 State Key Laboratory of Food Nutrition and Safety, School of Food Engineering and Biotechnology, Tianjin University of Science & Technology, Tianjin, 300457 China; 2 The University of Sydney, Sydney Institute of Agriculture, School of Life and Environmental Sciences, Sydney, NSW 2006 Australia; 3 Research center of Food Science and Human Health, School of Medicine, Nankai University, Tianjin, 300071 China

## Abstract

A thorough understanding of starch gelatinization is needed to control starch functional properties for food processing and human nutrition. Here, we reveal the mechanism of structural disassembly of rice, maize and wheat starch granules during thermal transitions in which a Rapid Visco Analyzer (RVA) was used to pre-heat the starches to certain transition points in the differential scanning calorimetry (DSC) heating profiles. This was done to generate sufficient material for structural analyses. The results from DSC, Raman, X-ray diffraction and scanning electron microscopy (SEM) analyses all showed that at the conclusion temperature (T_c_) of the DSC endotherm rice starch gelatinization was complete, whereas residual structural order remained in maize and wheat starches. Gelatinization of wheat and maize starch was complete at a temperature higher than T_c_ in the profile, which we define as the end temperature (T_e_). We propose that T_e_ would be better to define the completion point of starch gelatinization than T_c_.

## Introduction

Starch is a major component in many foods and the main glycemic carbohydrate in the human diet. Native starch granules are insoluble in cold water and are made up of two α-glucans: lightly branched amylose and highly branched amylopectin. Amylose is proposed to be more concentrated at the periphery^1,2^ or in the amorphous core area of the starch granules^3^, although some molecules may be interspersed radially among the amylopectin clusters. Starch granules have a complicated hierarchical structure, which is examined at nano to micrometer scales covering: glucosyl links (0.3 to 0.5 nm); alternating crystalline and amorphous lamellae with a periodicity of 9–11 nm; the “blocklet” structures (20 to 500 nm); the semicrystalline growth rings (120 to 550 nm); intact granules (1 to 100 μm)^4,5^. The multi-scale structures of granules and the changes they undergo during processing and storage are the major determinants of starch functionality and food quality^6,7^.

When an aqueous suspension of starch granules is heated above a certain temperature, the granules undergo an irreversible order-disorder transition known as gelatinization. Despite extensive studies, there is still a lack of a clear and agreed understanding of the mechanism of this transition^8–11^. Many features of gelatinization are incompletely understood, especially the nature of structural changes that give rise to the endo- and exotherms observed in Differential Scanning Calorimetry (DSC) thermograms. DSC is the most widely applied technique to measure heat of gelatinization of starches, but comparisons between studies on the extent of phase transitions are sometimes difficult due to considerable variations in experimental conditions, especially water content, heating rate and time^12,13^.

A combination of DSC with X-ray Diffraction (XRD) and Small Angle X-ray Scattering (SAXS) has shown that residual crystallinity and lamellar organization remain at the completion of the gelatinization endotherm^9,14^. Recent studies using DSC and swelling power measurements show that at water:starch ratios of 2:1 or greater, the gelatinization endotherm represents partial swelling of starch granules rather than full gelatinization^15,16^. To further understand the nature of the gelatinization endotherm obtained at high water contents, pre-heated wheat starch at different stages of gelatinization was generated using the Rapid Visco Analyzer (RVA) to simulate the DSC heating profiles^17^. The multi-scale structures of these gelatinized starches were characterized by a combination of XRD, Fourier Transform Infrared Spectroscopy (FTIR), Raman, SAXS, DSC and scanning electron microscopy (SEM), which showed that over a wide range of water content (0.5:1~4:1), considerable long- and short-range molecular orders remain at the conclusion temperature (T_c_, i.e., the intersection point on the baseline of a tangent to the part of the DSC trace beyond the peak temperature) of the starch gelatinization endotherm^18^.

**Figure 1:**
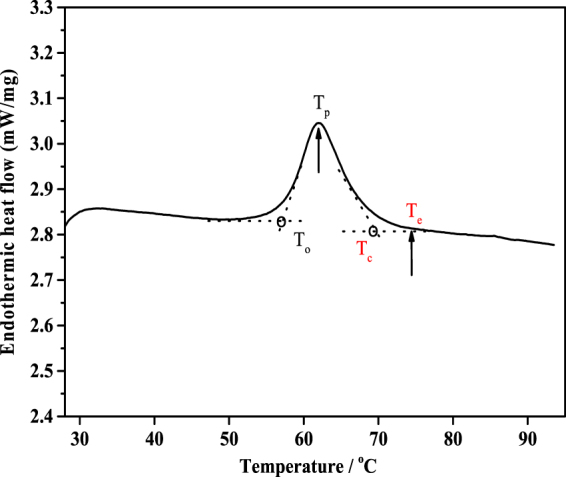
DSC thermogram of wheat starch at a water:starch ratio of 3.0:1 showing T_c_ and T_e_.

In previous studies, an RVA instrument was used without stirring as a model system to mimic the DSC heating profile and prepare sufficient materials to analyse starch structural changes during gelatinisation and retrogradation^17,18^. These studies showed that heating starch in the RVA without stirring in different water:starch mixtures of ratios ranging from 0.5 to 4.0 brought about a loss of granular organisation, although some long- and short-range structural order remained at the equivalent of the DSC conclusion temperature. These results, which are consistent with starch gelatinization having been shown to be incomplete after the DSC heating profile^15,16^, indicate the RVA is useful to model the DSC thermal transitions and provide new insights into structural changes that occur during gelatinisation and retrogradation of starch. In the present study, maize, rice and wheat starches were used to further reveal the nature of DSC endothermic transitions over a wide range of starch:water ratios with the aid of RVA heating. The multi-scale structural changes in these starches were examined after heating in the RVA to T_c_ and to a higher temperature (T_e_), which is defined as the point when the DSC trace reaches a flat baseline^15,16^, as shown in Fig. 1. To the best of our knowledge, this is the first time that the multi-scale structural changes were compared at T_c_ and T_e_ of DSC endotherm_._ Our results suggested that T_e_ would be better than T_c_ to define the point of completion of starch gelatinization.

## Materials and Methods

### Materials

Starch was isolated from wheat and rice grains as described previously^17,18^. The amylose contents of wheat and rice starches were 25.0 and 11.5%, respectively, as determined according to the method of Chrastil^19^. Non-waxy maize starch (27.0% amylose), and amylose (A0512) and amylopectin (A8515) from potato starch, were purchased from Sigma Chemical Co. (St. Louis, Mo., U.S.A.). All other chemical reagents were all of analytical grades.

### Differential scanning calorimetry

Thermal properties of starch samples were measured using a Differential Scanning Calorimeter (200F3, Netzsch, Germany) equipped with a thermal analysis data station. Approximately 3.0 mg of native starches were weighed accurately into an aluminum crucible. Distilled water was added to obtain water:starch ratios (v/w) of 0.5, 0.75, 1.0, 1.5, 2.0, 3.0 and 4.0 in the DSC pans, corresponding to water contents of 33.3, 42.9, 50.0, 60.0, 66.7, 75.0 and 80.0%, respectively. The sealed pans were allowed to stand at room temperature for 4 h before analysis. The pans were heated from 20 to 95 °C at a rate of 10 °C/ min. An empty aluminum pan was used as the reference. For the freeze-dried starch samples obtained from RVA, a ratio of water:starch of 5:1 (v/w) was used in the DSC measurements. The thermal transition parameters (onset (T_o_), peak (T_p_), conclusion (T_c_) temperatures and enthalpy change (ΔH)) were determined from the data recording software. The end temperature (T_e_), as defined in the introduction, was also determined (Fig. 1).

### Preparation of starch samples using a RVA instrument

To obtain sufficient material to characterize structural changes of starch during DSC heating, gelatinized starch samples were prepared by heating starch-water systems in a RVA instrument without stirring under the same heating profile described above for DSC measurements^17^. Approximately 2.5 g of native starches were weighed accurately into an RVA canister and distilled water was added with a pipette to obtain the water:starch ratios of 0.5, 0.75, 1.0, 1.5, 2.0, 3.0 and 4.0 (v/w). The mixtures in the canister were allowed to stand for 4 h at room temperature before heating. Rice and maize starch-water suspensions were heated separately in the RVA canisters without stirring to T_o_ − 10, T_o_, T_p_, T_c_, T_c_ + 10, 95 °C. Wheat and maize starch-water suspensions were also heated to T_e_ (Fig. 1). After heating to the designated temperature, the samples were quickly frozen in liquid nitrogen, freeze-dried, ground into a powder, and passed through a 100 μm sieve. A water:starch mixture (4:1, v/w) that was freeze-dried without prior heating was used as a control.

### Laser confocal Micro-Raman (LCM-Raman) spectroscopy

Raman spectra of freeze-dried starch samples were measured using a Renishaw Invia confocal Raman microscope system (Renishaw, Gloucestershire, United Kingdom). A 785 nm green diode laser source was used in this study. Spectra were obtained from at least five different positions of each sample in the range of 3200–100 cm^−1^. The full width at half maximum (FWHM) of the band at 480 cm^−1^ is used to characterize the short-range molecular order of starch samples^20,21^.

### X-ray diffraction

X-ray diffractograms were obtained using an X-ray diffractometer (XRD-6100, Shimadzu, Tokyo, Japan) with a Cu-K_α_ source (λ = 0.154 nm) running at 40 kV and 30 mA. All starch samples were stored for one week in a desiccator over a saturated NaCl solution before measurements. The samples were scanned from 4° to 35° (2θ) at a speed of 2°/min and a step size of 0.02°. The relative crystallinity was quantified as the ratio of the crystalline area to the total area between 4° and 35° (2θ) using the Origin software (Version 8.0, Microcal Inc., Northampton, MA, USA).

### Scanning electron microscopy (SEM)

The morphology of starch samples was observed using a scanning electron microscope (JSM-IT300LV, JEOL, Japan). Freeze-dried starch samples were mounted on a stub using double-sided adhesive tapes, sputter coated with gold in a sputter coater (JEC-3000FC, Tokyo, Japan). The imaging was performed at an accelerating voltage of 10 kV.

### Statistical analysis

Results are reported as the means and standard deviations of at least duplicate measurements. Analyses of variance (ANOVA) by Duncan’s test (p < 0.05) were conducted using the SPSS 19.0 Statistical Software Program (SPSS Inc. Chicago, IL, USA).

## Results and Discussion

### Thermal properties of native rice and maize starches

The thermal transition parameters of rice and maize starches are presented in Table 1. These two starches displayed gelatinization transitions in the temperature ranges 58.9–72.4 °C (rice) and 64.3–77.2 °C (maize) over a wide range of water contents. With increasing water content, the gelatinization transitions became progressively more pronounced and symmetrical (thermograms not shown). The ∆H of rice starch increased from 2.5 to 12.3 J/g as the water:starch ratio increased from 0.5 to 3, whereas for maize starch ∆H increased from 1.1 to 11.8 J/g with increasing water content from 0.5 to 2.0. The thermal transition temperatures of rice starch did not change greatly with increasing water content. However, T_c_ of maize starch increased from 72.5 to 77.2 °C as water:starch ratio increased from 0.5 to 2.0, above which it increased slightly. The effect of water content on thermal properties of starch has been studied extensively; the extent of crystallite melting is limited at low water content but increases with increasing water availability^11,12,22^. From this and other previous studies, it can be seen that the source of starch is a major determinant of its thermal properties^9,10,15,23^.

**Table 1:** Thermal transition parameters of native rice and maize starches.

Starch	Water:starch ratio (v/w)	T_o_ (°C)	T_p_ (°C)	T_c_ (°C)	∆H (J/g)
Rice	0.5	59.7 ± 0.0ab	65.6 ± 0.2ab	71.6 ± 0.1bc	2.5 ± 0.4e
	0.75	59.3 ± 0.3bc	64.8 ± 0.1c	70.0 ± 0.2de	5.7 ± 0.3d
	1.0	58.9 ± 0.2d	64.9 ± 0.2c	69.6 ± 0.2e	5.3 ± 0.2d
	1.5	60.0 ± 0.2a	65.2 ± 0.0bc	70.2 ± 0.2d	10.0 ± 0.2c
	2.0	59.2 ± 0.1 cd	65.2 ± 0.0bc	71.4 ± 0.4c	11.2 ± 0.0b
	3.0	59.8 ± 0.1a	65.8 ± 0.2a	72.4 ± 0.1a	12.3 ± 0.6a
	4.0	59.6 ± 0.1abc	65.4 ± 0.1ab	71.8 ± 0.1b	12.8 ± 0.0a
Maize	0.5	64.4 ± 0.2d	68.7 ± 0.2e	72.5 ± 0.4d	1.1 ± 0.2e
	0.75	64.3 ± 0.2 cd	70.2 ± 0.1 cd	74.6 ± 0.3c	3.6 ± 0.0d
	1.0	64.6 ± 0.2c	70.0 ± 0.2d	74.8 ± 0.1c	6.2 ± 0.4c
	1.5	65.1 ± 0.1b	70.3 ± 0.1c	76.3 ± 0.2b	11.4 ± 0.1ab
	2.0	65.3 ± 0.1b	70.8 ± 0.1b	77.2 ± 0.2a	11.8 ± 0.4a
	3.0	65.8 ± 0.1a	71.1 ± 0.2ab	76.1 ± 0.3b	11.4 ± 0.4ab
	4.0	65.6 ± 0.1a	70.8 ± 0.1a	76.6 ± 0.4b	10.8 ± 0.5b

Values are means ± SD. Values with the same letters in the same column are not significantly different (p < 0.05).

### Thermal properties of rice and maize starches pre-heated in the RVA

**Figure 2:**
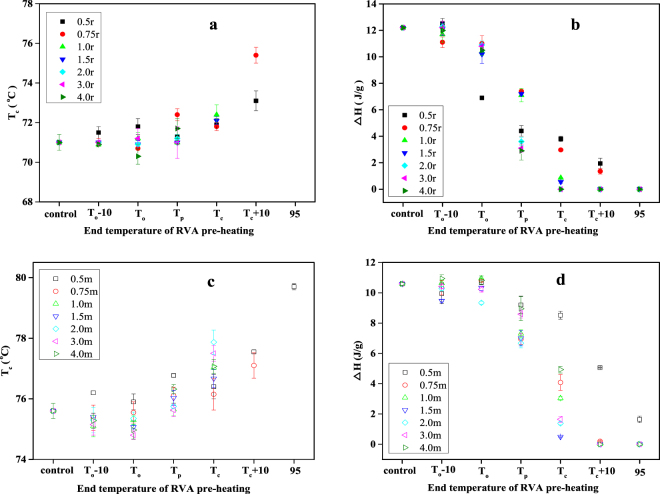
Thermal transition parameters of starch samples after pre-heating to different temperatures in RVA canisters. T_c_ of rice (**a**) and maize (**c**) starch samples; ∆H of rice (**b**) and maize (**d**) starch samples.

A typical DSC endothermic transition was observed for both starches after they were pre-heated in the RVA at a water:starch ratio of 0.5, with the exception of rice starch pre-heated to 95 °C. However, no endothermic transitions were observed for both starches after pre-heating a water:starch mixture of 0.75:1 in the RVA to 95 °C, nor after pre-heating water:starch mixtures of 1:1 or greater to T_c_ + 10 or 95 °C. With water-starch mixtures of 2:1, 3:1 and 4:1, endothermic DSC transitions were observed for maize starch after pre-heating to T_c_ in the RVA, but none were observed for rice starch. The values of T_c_ for rice and maize starches increased as the temperature of RVA pre-heating was increased (Fig. 2a,c). Similar results were obtained in a previous study on wheat starch^17,24^.

The enthalpy change (∆H) reflects primarily the disruption of long-range starch crystallinity during DSC heating^17^. The ∆H of all starch samples decreased markedly with increasing temperature of RVA pre-heating (Fig. 2b,d), indicative of the gradual disruption of starch crystallites during heating in the presence of water. As the water:starch ratio was 2:1 or greater, there was no measurable enthalpy change for the rice starch samples, indicating that starch crystallites were completely disrupted. However, values of ∆H were obtained for the maize starch samples pre-heated to T_c_ in water-starch mixtures of 2:1 or greater.

### Short- and long-range structural order of rice and maize starch

**Figure 3:**
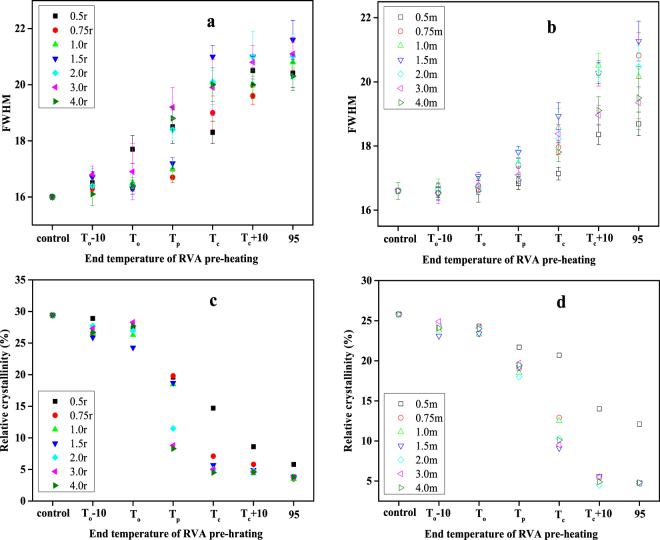
The FWHM values of rice (**a**) and maize (**b**) starch samples; the relative crystallinity of rice (**c**) and maize (**d**) starch samples.

The changes in short- and long-range structural order of the two starches during DSC heating, as modelled in the RVA, were monitored by Raman, and XRD spectra. The intensity of the Raman bands decreased progressively with increasing end temperature of RVA pre-heating (Figure not shown). The full width at half maximum (FWHM) of the band at 480 cm^−1^ of all rice (Fig. 3a) and maize starch samples (Fig. 3b) increased with increasing end temperature of RVA pre-heating. It is interesting to note that the FWHM for maize starch did not change greatly with RVA pre-heating to T_p_, but increased steadily from T_p_ to 95 °C, indicating that there was some short-range structural order at T_c_, the conclusion point of thermal transitions. For rice starch, there was a small increase in FWHM after RVA pre-heating to T_p_, and FWHM continued to increase as the temperature of RVA pre-heating was raised to T_c_, above which it remained essentially unchanged.

All of the water:starch mixtures presented decreasing relative crystallinity with increasing end temperature of RVA pre-heating (Fig. 3c,d). After pre-heating water:rice starch mixtures of 1:1 or higher to T_c_, the relative crystallinity was very low (about 5%), indicating that at T_c_ of the gelatinization endotherm, crystallites in rice starch were essentially all disrupted. However, with all maize starch samples after pre-heating to T_c_ in the RVA, the typical A-type X-ray diffraction patterns could still be observed, with residual crystallinity of 9 to 21% (Fig. 3d). The relative crystallinity of maize starch only decreased sharply with pre-heating to temperatures above T_p_.

### Granular morphology of starch samples

**Figure 4:**
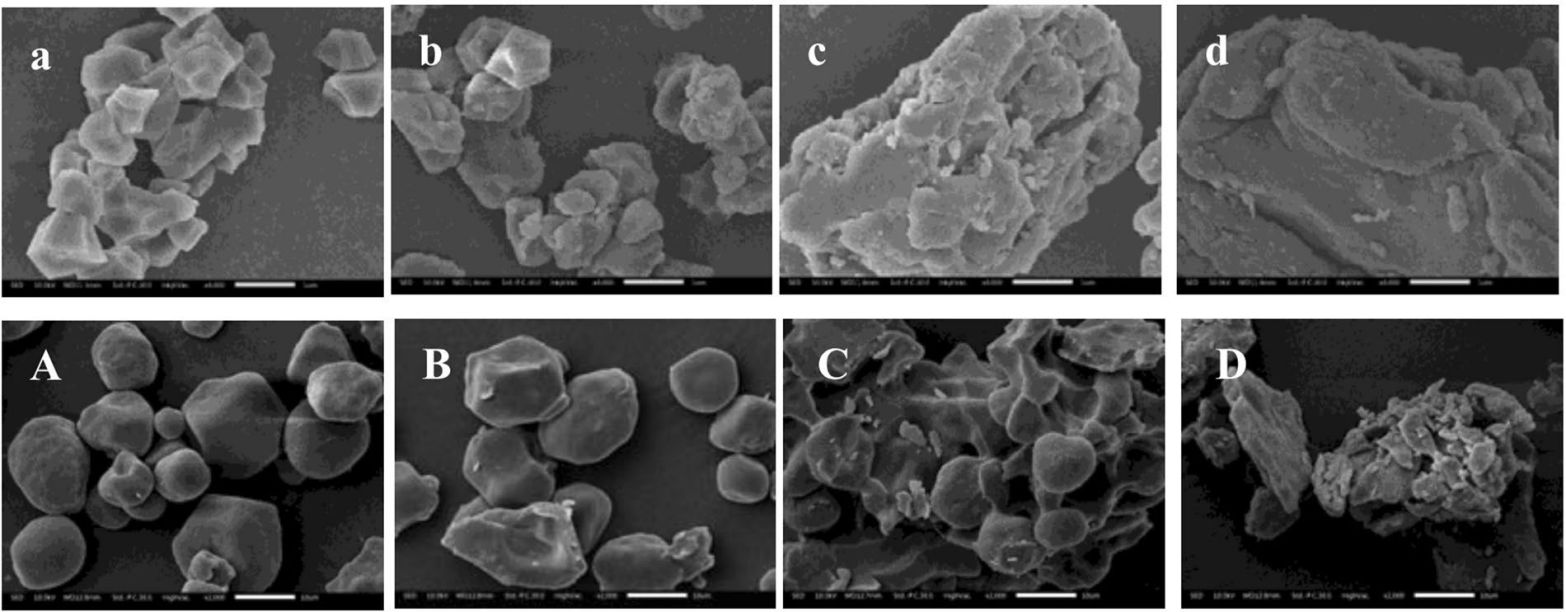
SEM images of rice and maize starch-water mixtures of 1:2 after pre-heating to different temperatures. (**a**) rice starch pre-heated to T_o_, (**b**) rice starch pre-heated to T_p_, (**c**) rice starch pre-heated to T_c_, (**d**) rice starch pre-heated to 95 °C. (**A**) maize starch pre-heated to T_o_, (**B**) maize starch pre-heated to T_p_, (**C**) maize starch pre-heated to T_c_, (**D**) maize starch pre-heated to 95 °C. Magnification, 4000× (rice starch), 2000× (maize starch).

SEM micrographs of rice and maize starch samples at a water:starch ratio of 2:1 after pre-heating to different temperatures are shown in Fig. 4. No significant differences were noted in granular morphology between native starch and starch samples after pre-heating to T_o_ (Fig. 4a,A). After pre-heating to T_p_, many rice starch granules were deformed and disrupted (Fig. 4b), whereas maize starch showed fewer changes in granular morphology (Fig. 4B). There were still clear granules in maize starch samples after pre-heating to T_c_ (Fig. 4C), while no intact starch granules were observed in rice starch samples (Fig. 4c). All granules were disrupted after pre-heating to 95 °C (Fig. 4d,D). The morphology changes of both rice and maize starches were generally consistent with DSC, and Raman results.

**Figure 5:**
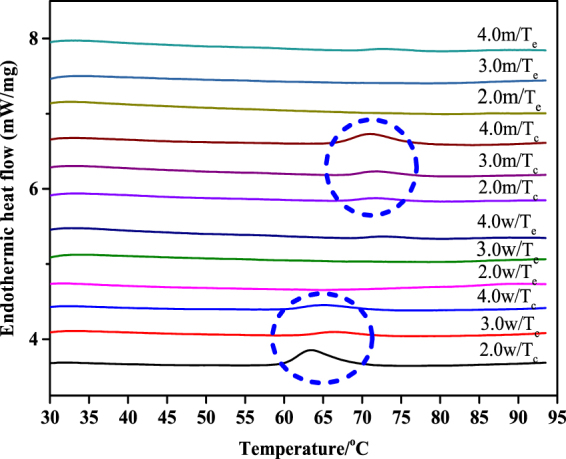
DSC thermograms of wheat (**a**) and maize (**b**) starch samples after pre-heating to T_c_ and T_e_. The blue circles indicated small endotherm after pre-heating to T_c_ was still present.

From the present study and our previous study^17^, we found that gelatinization of rice starch at a water:starch ratio of 2:1 or greater was complete at T_c_ of DSC thermal transition, whereas this was not observed for maize and wheat starches. Hence, further experiments were performed to define when complete gelatinization of these starches occurs. Accordingly, wheat and maize starch samples were prepared by pre-heating water:starch mixtures of 2.0 to 4.0 in the RVA to T_c_ and T_e_. Thermal properties of wheat and maize starch samples after pre-heating to T_c_ and T_e_ were noticeably different (Table 2). After pre-heating to T_c_, there were significant enthalpy changes of both starch samples on subsequent heating in the DSC, with typical DSC endothermic transitions being observed (Fig. 5, blue circle). However, enthalpy changes were not detected for both starches after pre-heating to T_e_.

**Table 2:** Enthalpy changes of wheat and maize starch samples after pre-heating to T_c_ and T_e._

Starch	Water/starch ratio (v/w)	T_c_ (°C)	∆H (J/g)	T_e_ (°C)	∆H (J/g)
Wheat	2.0	65.6 ± 0.1b	2.8 ± 0.1a	71.0 ± 0.1c	nd
	3.0	67.5 ± 0.4a	1.7 ± 0.2b	73.0 ± 0.0b	nd
	4.0	67.4 ± 0.5a	2.6 ± 0.1a	73.6 ± 0.2a	nd
Maize	2.0	77.2 ± 0.2a′	1.4 ± 0.1b′	82.1 ± 0.2a′	nd
	3.0	76.1 ± 0.3b′	1.7 ± 0.2b′	81.8 ± 0.1ab′	nd
	4.0	76.6 ± 0.4b′	2.9 ± 0.2a′	81.3 ± 0.1c′	nd

Values are means ± SD. Values with the same letters in the same column are not significantly different (p < 0.05). nd: not determined.

**Figure 6:**
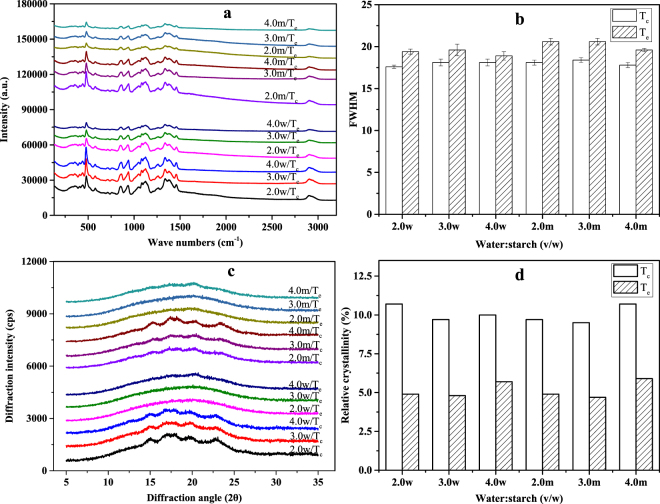
The LCM-Raman spectra (**a**) and FWHM values (**b**); the XRD diffraction patterns (**c**) and relative crystallinity (**d**) of wheat and maize starch samples.

The short- and long-range ordered structures after pre-heating to T_c_ and T_e_ were compared. Both starch samples presented similar Raman and XRD spectra (Fig. 6a,c). The intensity of the Raman bands decreased significantly for both starches pre-heated to T_e_ compared with pre-heated to T_c_ (Fig. 6a), indicating greater disruption of starch crystallites after pre-heating to T_e_ than to T_c_. The FWHMs (Fig. 6b) of water-starch mixtures of 2:1 to 4:1 that were preheated to T_e_ was higher than to T_c_.

The diffractograms of water-starch mixtures of 2:1 to 4:1 after pre-heating to T_c_ and T_e_ differed significantly (Fig. 6c). After pre-heating to T_c_, all starch samples showed the typical A-type X-ray diffraction patterns, whereas the diffraction peaks almost disappeared after pre-heating to T_e_. The relative crystallinity was lower for samples pre-heated to T_e_ than for samples pre-heated to T_c_ (Fig. 6d), consistent with the results of Raman and DSC. These observations indicated that greater disruption of starch ordered structures at T_e_ occurred than at T_c_.

Structural disruption of rice starch commenced at the onset temperature of the gelatinization endotherm and was essentially completed at the conclusion temperature. During the gelatinization endotherm, multiscale structures in rice starch were disrupted progressively with increasing temperature from T_o_ to T_c_. From this result, we can conclude that the gelatinization endotherm of rice starch obtained at a water:starch ratio of 2:1 or greater represents the full gelatinization of rice starch. However, no structural order disruption occurred at T_o_ and some structural order in maize starch was still present at the conclusion temperature (T_c_) of the DSC endothermic transition obtained when the ratio of water:starch was 2:1 or greater. Further disruption of structural order occurred on heating beyond T_c_. This observation was consistent with the previous findings that residual crystallinity still remained at Tc^9,14,17^. Hence, we can conclude that gelatinization of maize and wheat starches did not commence at T_o_ and was not complete at T_c_ of the gelatinization endotherm. After pre-heating to T_e_, the multiscale structures of wheat and maize starches were completely disrupted, indicating a complete gelatinization at T_e_. Hence, we propose that T_e_ of starch gelatinization endotherm can be defined as conclusion temperature of DSC gelatinization endotherm.

The mechanism of the molecular disassembly of starch granules during DSC thermal transition has attracted much research attention^11,25^. At low to intermediate water content (<67% water; i.e., water starch mixtures of 2:1 or lower), the gelatinization endotherm represents limited swelling of starch granules rather than full gelatinization of starches^15–17^. In water:starch mixtures of 2:1 or higher, the gelatinization endotherm determined by DSC has been considered to represent complete gelatinization of starches since pioneering worked of Donovan^22^. However, more recent studies have provided evidence that even at a high water content in water:starch mixtures (>67% or water:starch ratio of 2:1), the gelatinization endotherm of wheat and pea starches does not represent full gelatinization^16,17,26^.

## Conclusions

In the present study, the extent of structural changes in starch at temperatures corresponding to key transition points in the DSC endotherm was investigated. To this end, an RVA was used without stirring to mimic DSC heating profiles and obtain sufficient amounts of starch for subsequent analyses by DSC, Raman, XRD and SEM. For rice starch, there was essentially no residual structural order remaining in water-starch mixtures of 2:1 to 4:1 after heating to T_c_, indicating the endotherm represents the complete gelatinization. However at T_c_, maize and wheat starches still had appreciable amounts of long- and short-range structural order, indicative of incomplete gelatinization. Further disruption of ordered structures occurred when wheat and maize starches were heated beyond T_c_ to T_e_, the temperature at which the DSC trace reaches a flat baseline. Hence, we conclude that gelatinization of wheat and maize starches starts after T_o_ and is more complete at T_e_ in water:starch mixtures of 2:1 or greater. As starch gelatinization properties are often measured in such mixtures, we propose that T_e_ can be defined as the end point of complete gelatinization.
