# Author Correction: New insights into gelatinization mechanisms of cereal endosperm starches

**DOI:** 10.1038/s41598-018-24180-x

**Published:** 2018-04-16

**Authors:** Shujun Wang, Chen Chao, Fengjuan Xiang, Xiu Zhang, Shuo Wang, Les Copeland

**Affiliations:** 1 State Key Laboratory of Food Nutrition and Safety, School of Food Engineering and Biotechnology, Tianjin University of Science & Technology, Tianjin, 300457 China; 2 The University of Sydney,Sydney Institute of Agriculture, School of Life and Environmental Sciences, Sydney, NSW 2006 Australia; 3 Research center of Food Science and Human Health, School of Medicine, Nankai University, Tianjin, 300071 China

Correction to: *Scientific Reports* 10.1038/s41598-018-21451-5, published online 14 February 2018

The original version of this Article listed incorrect affiliations for Shuo Wang and Les Copeland. The correct affiliations are listed below:

Shuo Wang: Research center of Food Science and Human Health, School of Medicine, Nankai University, Tianjin, 300071, China.

Les Copeland: The University of Sydney, Sydney Institute of Agriculture, School of Life and Environmental Sciences, Sydney, NSW, 2006, Australia.

In addition, the footnote of the PDF version of this article contained a typographical error in the spelling of the author Shuo Wang, which was incorrectly given as Shou Wang.

These errors have now been corrected in the HTML and PDF versions of this Article.

